# One-Pot Facile Synthesis of CuO–CdWO_4_ Nanocomposite for Photocatalytic Hydrogen Production

**DOI:** 10.3390/nano12244472

**Published:** 2022-12-16

**Authors:** Shaeel Ahmed Althabaiti, Maqsood Ahmad Malik, Manoj Kumar Khanna, Salem Mohamed Bawaked, Katabathini Narasimharao, Soad Zahir Al-Sheheri, Bushra Fatima, Sharf Ilahi Siddiqui

**Affiliations:** 1 Chemistry Department, Faculty of Sciences, King Abdulaziz University, P.O. Box 80203, Jeddah 21589, Saudi Arabia; 2 Department of Physics, Ramjas College, University of Delhi, Delhi 110007, India; 3 Department of Chemistry, Jamia Millia Islamia, Delhi 110025, India; 4 Department of Chemistry, Ramjas College, University of Delhi, Delhi 110007, India

**Keywords:** nanocomposites, photocatalysis, hydrogen production, sacrificial reagents, synergistic effect

## Abstract

Hydrogen (H_2_) is a well-known renewable energy source that produces water upon its burning, leaving no harmful emissions. Nanotechnology is utilized to increase hydrogen production using sacrificial reagents. It is an interesting task to develop photocatalysts that are effective, reliable, and affordable for producing H_2_ from methanol and acetic acid. In the present study, CuO, CdWO_4_, and CuO–CdWO_4_ nanocomposite heterostructures were prepared using a cost-efficient, enviro-friendly, and facile green chemistry-based approach. The prepared CuO, CdWO_4_, and CuO–CdWO_4_ nanocomposites were characterized using X-ray diffraction pattern, Fourier-transform infrared spectroscopy, diffuse reflectance ultraviolet–visible spectroscopy, scanning electron microscopy, transmission electron microscopy, selected area electron diffraction (SAED) pattern, N_2_ physisorption, photoluminescence, and X-ray photoelectron spectroscopy techniques. The synthesized photocatalysts were utilized for photocatalytic H_2_ production using aqueous methanol and acetic acid as the sacrificial reagents under visible light irradiation. The influence of different variables, including visible light irradiation time, catalyst dosage, concentration of sacrificial reagents, and reusability of catalysts, was studied. The maximum H_2_ was observed while using methanol as a sacrificial agent over CuO–CdWO_4_ nanocomposite. This enhancement was due to the faster charge separation, higher visible light absorption, and synergistic effect between the CuO–CdWO_4_ nanocomposite and methanol.

## 1. Introduction

As the world’s usage of fossil fuels increases at an alarming and unsustainable rate, the emission of greenhouse gases and other hazardous pollutants is rising to unacceptably high levels [1]. Oxides of nitrogen (NOx), sulfur (SOx), and suspended particulate matter that cannot be removed by filtration are the breath-taking poisons released by fuel combustion [2]. In addition, fossil fuels generate a large quantity of greenhouse gases, mostly carbon dioxide (CO_2_), and unfavorable byproducts in our ecosystem [3,4]. To overcome these disadvantages, alternative energy sources which are renewable and ecologically benign are essential for the long-term sustainable growth of society. Future energy sources must satisfy the requirements of releasing environmentally acceptable byproducts, renewability, and availability [1,4]. Nanotechnology is an emerging sophisticated technology in the realm of material science that could offer different options for the development of ecologically friendly renewable energy sources. Hydrogen (H_2_) is one of these attractive possible candidates, and the generation of H_2_ from renewable sources does not result in the emission of CO_2_. In this context, H_2_ can be considered a clean energy carrier that has the potential to solve currently existing problems in the areas of energy and environment [5,6,7]. H_2_ can be produced using renewable resources across the globe to minimize the need for fossil fuels. It is mostly used in the production of various chemicals, including ammonia and methanol, as well as in oil refineries to separate crude oil into lighter fractions [8].

A promising method for producing sustainable H_2_ is solar photocatalysis [6,7], which splits water into H_2_ gas [9,10]. Recently, Zn- and Ni-Co-doped hematite nanorods [5] and Ni_2_P–Co_2_P [6] photocatalysts were reported to be in use for the process of H_2_ production. It is also recognized that the photocatalysts based on semiconductor heterojunctions could enhance water splitting [10]. Many photocatalysts, including noble metal deposited oxides, metallic sulphides and oxynitrides, have also been utilized for H_2_ production, although these catalysts show poor stability [7]. Notably, water splitting is completed with two half-reactions (redox reaction), in which mainly four different steps are involved to tune the photocatalyst [10], including (i) light harvesting, (ii) charge excitation, (iii) charge separation and transfer, and (iv) surface electro-catalytic reaction. To make light harvesting smooth, surface modifications, such as anchoring functional groups, generating crystal defects, and enhancing surface area, could be performed [7,8,9,10]. In the second step, it is necessary to decrease the bandgap for the electron (e^−^) to be easily excited from the valence band (VB) to the conduction band (CB). When an e^−^ is excited from VB, it forms a positive hole (h^+^) which can be easily recombined, suggesting that it is necessary to stop the recombination of the e^−^ and the h^+^. In the third step, it is essential to prevent the recombination or decrease the recombination rate, where the interfacial electric field can be used through the generation of a heterojunction photocatalyst. In the fourth step, the e^−^ and h^+^ move to the surface of the semiconductor and execute water splitting through two-half (redox) reactions. It should also be noted that the surface redox reaction takes place only when the reduction and oxidation potentials are more positive and negative than the CB and VB levels, respectively [10].

Numerous photocatalysts with suitable characteristics have been developed to produce H_2_ using aqueous solutions of sacrificing agents [11,12,13]. To improve the photocatalytic performance and stability, different synthetic strategies, such as doping with noble metals and heterojunctions of different metal and metal oxide nanomaterials, are commonly adopted [14,15,16,17]. Despite the advances in photon-responsive applications of heterojunction photocatalysts, the photocatalysts are still suffering from several drawbacks such as visible light harvesting, charge carrier excitation, and charge separation. New efforts are being made to overcome the drawbacks of photocatalytic production of H_2_ through water splitting. One of the interesting approaches is to develop a simple, inexpensive, and sustainable green method to develop a stable and highly efficient visible-light-active photocatalyst for H_2_ production.

CuO is a p-type semiconductor with a narrow bandgap (Eg = 1.2–1.6 eV), which offers a wide range of applications, including optical, electrical, catalytic, and photocatalytic degradation, as well as serves as an antioxidant and an adsorbent [18,19,20,21,22]. Due to the distinctive physical and chemical characteristics of CuO, CuO nanoparticles (NPs) have been used in catalytic processes, chemical sensors, antimicrobial textiles, and batteries [23,24,25]. On the other hand, cadmium tungstate (CdWO_4_) exhibits superior chemical, optical, structural, and thermal properties [26,27]. The broad bandgap (3.8 eV) of CdWO_4_ restricts its use in the solar spectral response range [28]. The advantages of combining CdWO_4_ nanomaterials into composites can be fully exploited by forming heterojunction [29,30]. The extract of the *Brassica rapa* plant was used for the synthesis of functionalized CuO and CdWO_4_ samples as the *Brassica rapa* plant extract contains several phytochemicals (such as quercetin, kaempferol, isorhamnetin, anthocyanins, and derivatives of cinnamic acid), which can act as capping, stabilizing, and functionalizing agents for nanocomposites [22]. This synthesis protocol could be considered as a sustainable green method due to the usage of water as a solvent, low energy requirement, cost effectiveness, and the offer of a high yield of products [22]. In addition to the above-mentioned advantages, *Brassica rapa* plant extract could introduce different functional groups, such as -COOH and -CHO, onto the surface of the metal oxides. These surface functional groups could assist to enhance the absorption of light and improve the photocatalytic activity [31]. Akhtar et al. [32] and Narayanan et al. [33] synthesized silver nanoparticles using *Brassica rapa* leaf extract and utilized the synthesized materials as antimicrobial agents. 

In our previous reports, we synthesized CuO [22] and CdWO_4_ [27] by utilizing *Brassica rapa* leaf extract. In this contribution, bare CuO, CdWO_4_, and CuO–CdWO_4_ nanocomposite samples were synthesized by a cost-efficient, enviro-friendly, and facile green chemistry-based approach involving *Brassica rapa* leaf extract. The prepared CuO, CdWO_4_, and CuO–CdWO_4_ nanocomposite samples were characterized using X-ray diffraction pattern, Fourier-transform infrared spectroscopy, diffuse reflectance ultraviolet–visible spectroscopy, scanning electron microscopy, transmission electron microscopy, selected area electron diffraction (SAED) pattern, N_2_ physisorption, photoluminescence, and X-ray photoelectron spectroscopy techniques. The synthesized photocatalysts were utilized for photocatalytic H_2_ production using aqueous methanol and acetic acid as the sacrificial reagents under visible light irradiation.

## 2. Experimental Details

### 2.1. Materials

Analytical grade copper acetate monohydrate (199.6 g/mol), cadmium iodide (366.2 g/mol), and sodium tungstate dihydrate (329.8 g/mol) were obtained from Merck Ltd. (Rahway, NJ, USA) and utilized without purification for the preparation of the photocatalysts. Fresh *Brassica rapa* leaves were procured from the local market.

### 2.2. Preparation of Plant Extract

Fresh *Brassica rapa* leaves were washed initially with tap water and then with double distilled water several times. The leaves were then dried in an electric oven at 60 °C for 48 h. Then, the fine powder of the leaves was obtained by grinding the dried leaves. The collected powder was then used to prepare the aqueous extract by adding 20 g of powder in 200 mL of doubled distilled water, and then the water was heated at 70 °C for 90 min. Finally, the extract was separated from the soaked powder using vacuum filtration. The obtained extract was utilized for the synthesis of the nanocomposites.

### 2.3. One-Pot Synthesis of CuO–CdWO_4_ Nanocomposite

The parent metal oxides (CuO and CdWO_4_) were prepared by using aqueous *Brassica rapa* leaf extract following a previously reported method [22,27]; the detailed information is provided in the supplementary information. The CuO–CdWO_4_ nanocomposite was synthesized using aqueous *Brassica rapa* leaf extract through a one-pot facile route. Initially, a salt solution of Cu, Cd, and W was obtained by mixing 300 mL of 0.05 M Na_2_WO_4_ solution, 100 mL of 0.05 M CdI_2_ solution, and 100 mL of 0.4 M Cu(COOCH_3_)_2_ solution. The mixture was stirred for 30 min to obtain a clear homogeneous solution. Then, 160 mL of prepared *Brassica rapa* leaf extract and 200 mL of 1 M of NaOH solution were added and the mixture was stirred at 60 °C for 1 h. This led to the change in the color of the solution from brown to blue, which indicated the formation of a nanocomposite. The blue-colored solution was further continuously stirred at 100 °C for 3 h to achieve complete nanocomposite formation. The obtained precipitate was washed several times with distilled water, filtered, and dried in an oven at 60 °C for 24 h. The dried sample was grounded to make a powder and was calcined at 450 °C for 5 h.

### 2.4. Characterization of Materials

The X-ray diffraction (XRD) measurements were performed using a Philips PW-3710 diffractometer equipped with Cu K_α_ radiation. The diffuse reflectance ultraviolet-visible (UV-*vis*) spectrum of the powder sample was collected using a Lambda 950 PerkinElmer UV-*vis* spectrophotometer. The morphology of the materials was characterized using a scanning electron microscope (SEM, JSM-6510, Joel, Japan) equipped with an energy-dispersive X-ray spectroscope (EDX, Bruker 127 eV). A JEM-2100 transmission electron microscope (TEM) was used to obtain the TEM images and the particle size distribution of the prepared nanocomposites. Fourier-transform infrared spectroscopy (FT-IR) spectrum was measured by using a Perkin-Elmer full-range FTIR spectrometer in the range of 4000–400 cm^−1^. Selected area electron diffraction (SAED) pattern was also analyzed to investigate the crystal lattice and structure of the synthesized materials. The N_2_ physisorption analysis of the samples was performed using a Micromeretics Tristar 2000 instrument. The samples were degassed under helium gas at 300 °C for 2 h prior to analysis. The photoluminescence (PL) spectra of the samples were measured using a fluorescence spectrophotometer (F-4500, Hitachi). The X-ray photoelectron spectroscopy (XPS) patterns for the synthesized samples were obtained using a Thermo-Scientific Escalab 250 Xi XPS instrument with Al Kα X-ray having a spot size of 650 mm. The peak shift due to charge compensation was corrected using the C*1s* binding energy.

### 2.5. Hydrogen Production Activity

**Scheme 1:**
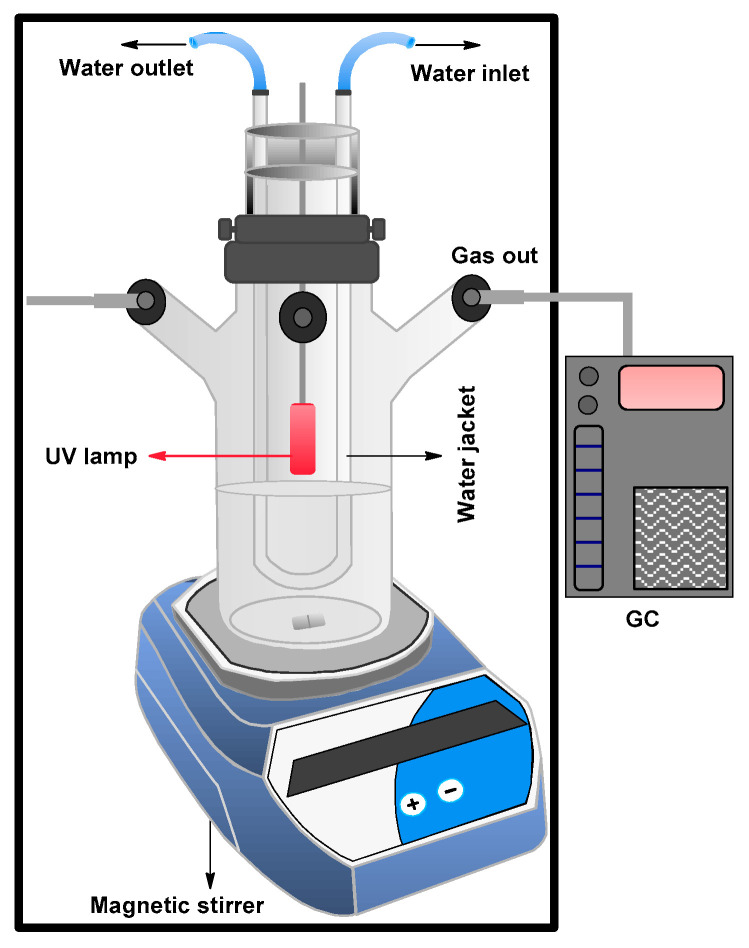
Schematic representation of the photocatalytic reactor for H_2_ production.

The photocatalytic H_2_ production experiments were performed in a photochemical reactor connected with a GC instrument. The reactor was a tightly closed glass reactor (total volume of 1000 mL) equipped with a cold-water circulation pump to reduce the heat generated during the photocatalytic reaction process. For each photocatalytic experiment, 100 mg of the synthesized catalyst powder was dispersed in a mixed solution of 400 mL DI water and sacrificial reagent (20 vol.% of methanol). Then, the solution was transferred into the reactor and purged with argon gas for 30 min in order to flush all the atmospheric gases. The empty headspace was kept constant at 500 mL for all reactions. When all gases were completely purged, the reactor was irradiated using a 300 W Xenon arc lamp without any UV cutoff filter. The amount of hydrogen from the reaction was determined by flowing argon as a gas carrier through the reactor in order to carry the generated gases for gas chromatography analysis (GC-1000). The measurement was performed every 30 min at a 5 h span of photoreaction. The photocatalytic reaction setup is shown schematically in Scheme 1.

## 3. Results and Discussions

### 3.1. FT-IR Spectroscopy

**Figure 1:**
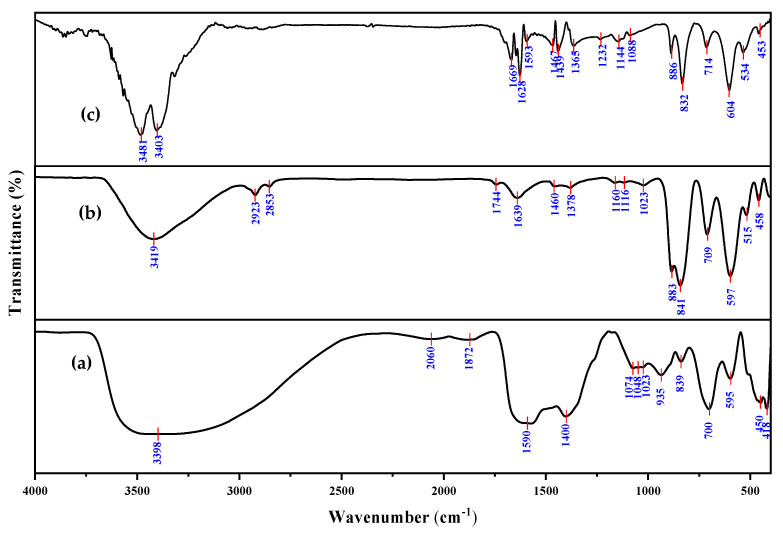
FT-IR spectra for (**a**) CuO (Reprinted with copyright permission from Fatima et al. [22]), (**b**) CdWO_4_ (Reprinted with copyright permission from Fatima et al. [27]), and (**c**) CuO-CdWO_4_ nanocomposite.

The FT-IR spectra of the CuO NPs, CdWO_4_ NPs, and CuO–CdWO_4_ NC samples are shown in Figure 1. The spectra show the presence of characteristic bands of functional groups (due to the plant extract) as well as metal-oxygen vibrations. The bands observed for the CuO NPs, CdWO_4_ NPs, and CuO–CdWO_4_ NC are listed in Table 1. Similar FT-IR spectra for CuO and CdWO_4_ samples were reported in the literature [22,27], which is explained in Appendix A. The characteristic IR absorption bands due to both CuO and CdWO_4_ phases are presented in the CuO–CdWO_4_ nanocomposite (Figure 1c). However, the position of the bands appears to be shifted because of the interaction between these two semiconductors compared to the individual spectra of CuO (Figure 1a) and CdWO_4_ (Figure 1b). The FT-IR spectrum of the CuO–CdWO_4_ nanocomposite reveals the bands around 3400 cm^−1^, pertaining to the stretching vibration of the O–H groups of adsorbed water or hydroxyl groups due to plant extract. The Cu–O bond has two bands at 604 cm^−1^and 714 cm^−1^ [34]. The other bands between 400–1000 cm^−1^ are the characteristics of the intrinsic vibrations from CdWO_4_ [35,36]. The stretching bands between 1700–1000 cm^−1^ are ascribed to various functional groups, such as -C=O, -NH, aromatic C=C, aromatic C−C, and -C−O groups, that came from the plant extract [22,27,32,37]. These results are in agreement with the previously reported literature [22,27,32,38].

### 3.2. XRD Diffraction

Powder X-ray diffraction technique was employed to confirm the crystal structure and phase purity of the bare CuO, CdWO_4_ oxides, and CuO–CdWO_4_ nanocomposite. The XRD pattern for bare CuO shows major reflections at 2θ values of 36°, 38°, 48°, 56°, and 64° corresponding to the (002), (111), (202), (020), and (113) planes of the monoclinic CuO phase [JCPDS file no. 45-0937]. Minor reflections due to the metallic Cu phase can also be seen in the XRD pattern that are assigned by the corresponding planes (111), (200), and (220) at 26.4°, 34.1°, and 70.10°, respectively. A similar results were observed in our previous publication [22].

**Figure 2:**
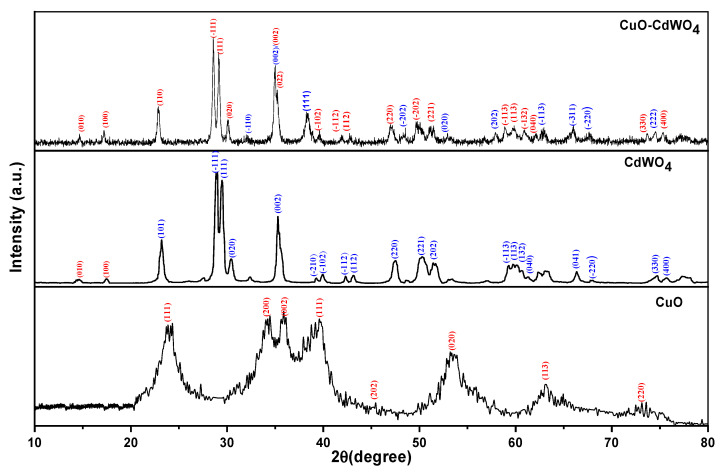
XRD patterns of CuO (Reprinted with permission from Fatima et al. [22]), CdWO_4_ (Reprinted with permission from Fatima et al. [27]), and CuO–CdWO_4_ nanocomposite.

The XRD pattern of the bare CdWO_4_ sample exhibits the characteristic reflections for the tetragonal CdWO_4_ crystal system with (101), (−111), (111), (002), (020), (112), (121), (103), (212), (−131), (311), (123), (213), and (014) planes [JCPDS card no. 80-0138], as shown in Figure 2. A similar result was reported for our previous publication [27]. The diffraction pattern of the CuO–CdWO_4_ nanocomposite (Figure 2) shows the characteristic reflections of both CuO (red marking) and CdWO_4_ (blue marking) NPs. The presence of intense sharp reflections indicates that the obtained nanocomposite is highly crystalline in nature. It has previously been reported that the CuO–CdWO_4_ composite contains a CuO (tenorite) crystal system with the monoclinic structure having cell dimensions, a = 4.6776 Å, b = 3.4593 Å, c = 5.1264 Å, and β = 98.965°, C12/c1 space group. (COD data Entry No. 96-901-5823) [39,40]. While the Scheelite CdWO_4_ crystal system with a tetragonal structure has cell dimensions, a = 5.1590 Å and c = 11.1690 Å (space group I41/a) (COD data Entry No. 96-154-9133) [40,41]. No deviation can be observed in the lattice parameters of the CuO–CdWO_4_ nanocomposite sample, indicating that the doping of any foreign metals into the crystal system did not occur. The average crystallite size of the CuO–CdWO_4_ nanocomposite was calculated using Scherrer’s formula, D = Kλ/βCosθ, where ‘D’ is the average crystal size, ‘K’ is the constant (0.89), ‘λ’ is the wavelength of the CuKα radiation, ‘β’ is the full width at half maximum, and ‘θ’ is the diffraction angle. The crystallite size of the CuO–CdWO_4_ nanocomposite was found to be around 25 nm higher than the bare CuO (10 nm) and CdWO_4_ (20 nm).

### 3.3. Morphological Studies

#### 3.3.1. SEM and EDX Analyses

**Figure 3:**
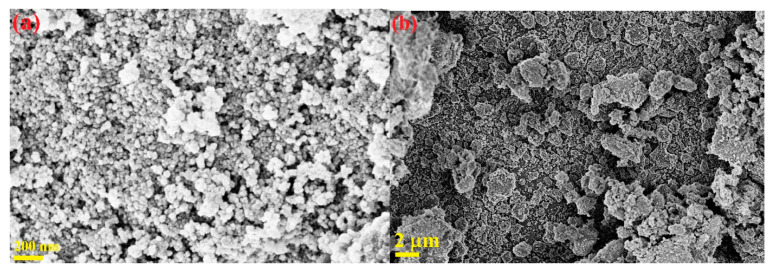

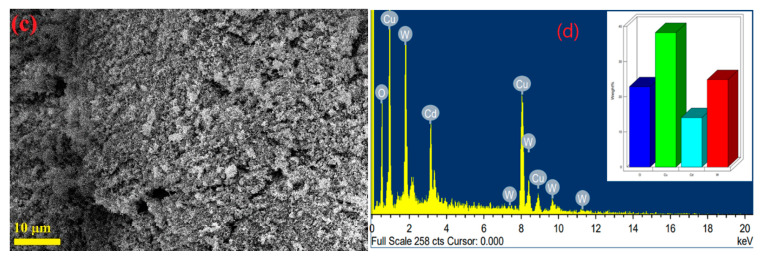
SEM images for (**a**) CuO, (**b**) CdWO_4_ and (**c**) CuO–CdWO_4_, and (**d**) EDX pattern for CuO-CdWO_4._

The SEM images of the CuO, CdWO_4_, and CuO–CdWO_4_ nanocomposite samples indicate the presence of irregularly shaped aggregated particles of various sizes with rough texture (Figure 3). The presence of macro-size pore channels in between the particle aggregates can be observed in the SEM image of the nanocomposite. The EDX analytical results for the CuO–CdWO_4_ nanocomposite are presented in Figure 3d and Table 2. The changes in the elemental composition in the CuO–CdWO_4_ composite from the parent CuO and CdWO_4_ can be clearly observed from the EDX analysis of the previously reported bare CuO [22] and CdWO_4_ [27] (Table 2). These elemental composition of the CuO–CdWO_4_ composite was found to be 22.85% O, 38.24% Cu, 14.05% Cd, and 24.85% W. These results suggest that successful one-pot synthesis of a CuO–CdWO_4_ composite is possible via the green method.

#### 3.3.2. TEM and SEAD Analyses

**Figure 4:**
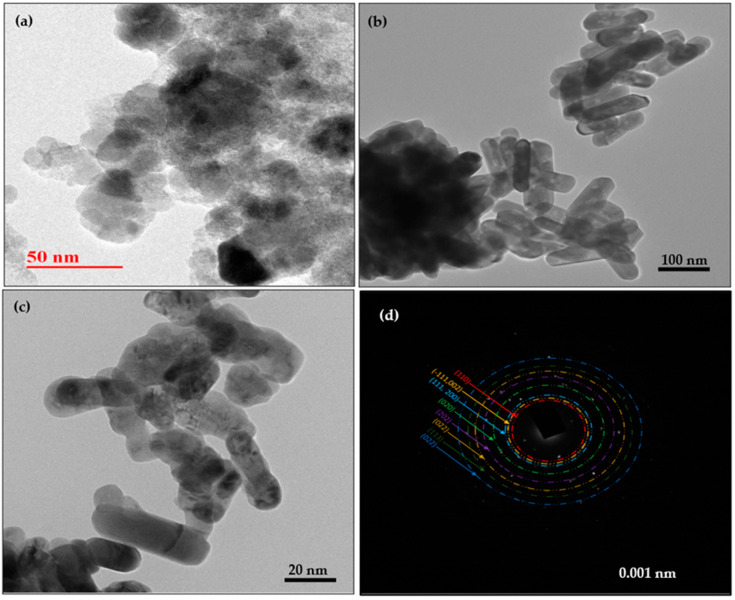
TEM images of (**a**) CuO, (**b**) CdWO_4_ and (**c**) CuO–CdWO_4_, and (**d**) SEAD pattern for CuO-CdWO_4_ nanocomposite.

The TEM analysis was performed to a get better understanding of the morphology and particle size of the synthesized samples. It is clear from the TEM images that the CuO crystals are grown with irregular shapes with small-size particles (Figure 4a), while the CdWO_4_ sample exhibits the presence of relatively large-size, rod-shaped particles (Figure 4b). Interestingly, the TEM image of the CuO–CdWO_4_ composite shows the presence of nanocapsules along with irregularly shaped agglomerated NPs, as shown in Figure 4c. The existence of an interaction between CuO and CdWO_4_ is confirmed by the oriented attachment between the metal oxides (rod-shaped CdWO_4_ and irregular CuO) [40,41]. The SEAD pattern for the CuO–CdWO_4_ sample is shown in Figure 4d; the image shows several diffraction rings corresponding to the planes for the CuO and CdWO_4_ crystal structures [41,42].

### 3.4. Nitrogen Physisorption Studies

**Figure 5:**
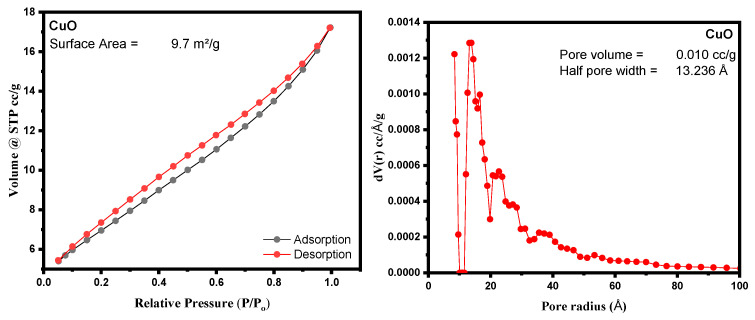

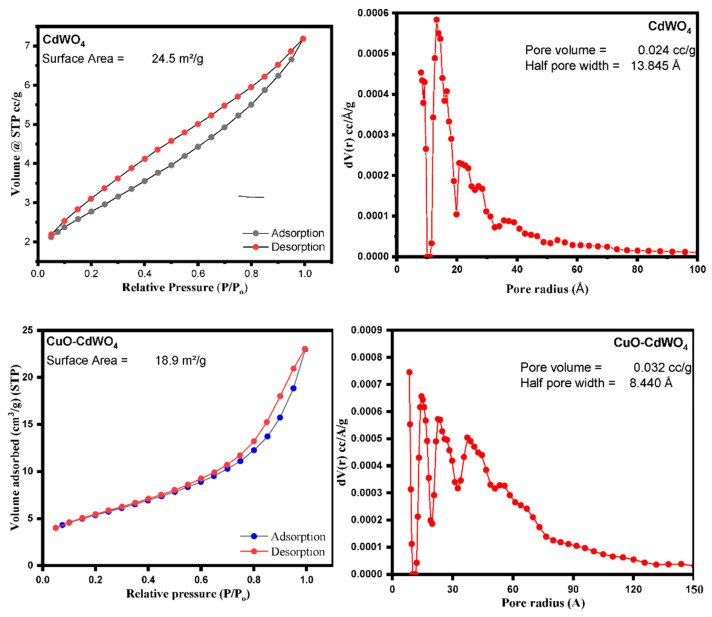
N_2_ adsorption-desorption isotherms and pore size distribution patterns for the CuO, CdWO_4_, and CuO–CdWO_4_ nanocomposite samples.

The textural properties for the synthesized CuO, CdWO_4_ and CuO–CdWO_4_ nanocomposite samples was investigated using N_2_ physisorption analysis (Figure 5). The N_2_ adsorption-desorption isotherms for the CuO, CdWO_4_, and CuO–CdWO_4_ samples adopt type-III isotherm with apparent H_3_ hysteresis loop as per IUPAC classification [43]. The occurrence of type-III adsorption-desorption isotherm reflects the interaction of the adsorbate on the surface of the adsorbent. The observed H_3_ hysteresis reflects N_2_ adsorption owing to the capillary condensation, and it is possibly due to the uneven distribution of aggregates in the sample with complex pore channels. The pore size distribution patterns for the synthesized samples were obtained using the NLDFT model. The results indicated the appearance of PSD peaks corresponding to macropores in the CuO–CdWO_4_ sample, probably due to the aggregation between CuO and CdWO_4_ particles forming large-size voids. The analysis reflected the specific surface area of the CuO–CdWO_4_, which was calculated to be 18.9 m^2^/g with a pore volume of 0.032 cc/g and a half pore width of 8.440 Å, respectively.

### 3.5. Determination of Optical Bandgap

**Figure 6:**
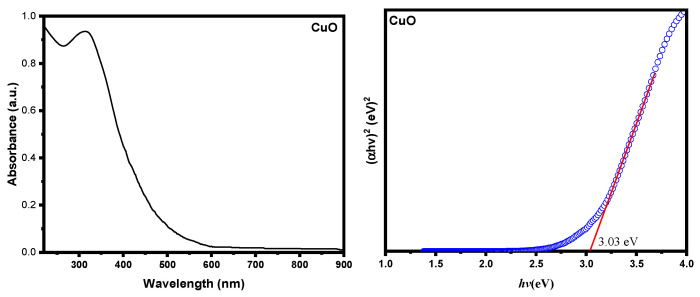

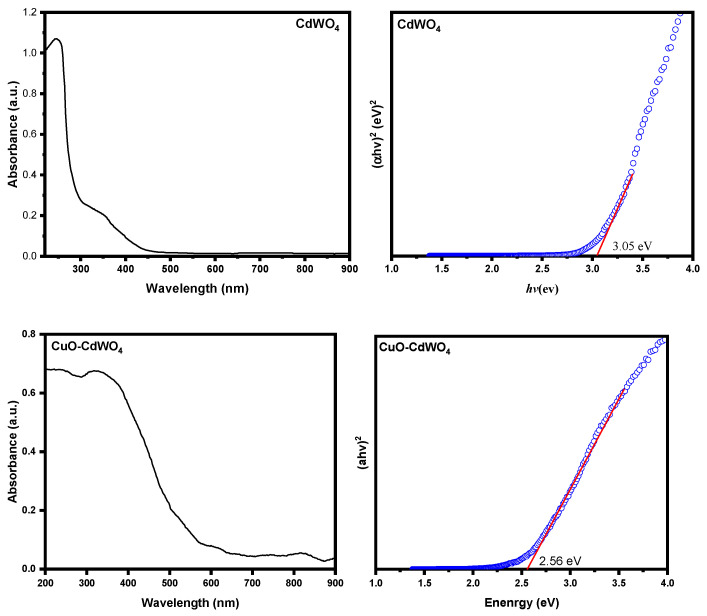
DR UV-*vis* spectra and the Tauc plots for CuO, CdWO_4_, and CuO–CdWO_4_ nanocomposite samples.

Figure 6 depicts the DR UV-*vis* absorption spectra for the CuO, CdWO_4_, and CuO–CdWO_4_ nanocomposite samples and shows a broad absorption peak centered around 350 nm. The optical absorption edge stretches from UV to visible region (from 300 nm to 500 nm), which may be attributed to the increase in the grain size of the samples. The UV-*vis* absorption data were used to measure the bandgap of the synthesized samples using the Tauc relation: (εhv)=C (hv−Eg)^2, where ‘C’ is a constant, ‘ε’ is a molar extinction coefficient, ‘Eg’ is the bandgap of the sample, and ‘n’ stands for evaluating the type of transition [44]. In the Tauc plot, the intercept of the linear portion of the (εhν)^2^ vs. hν is on the *x*-axis, as shown in Figure 6. The bandgap values for the CuO, CdWO_4_, and CuO–CdWO_4_ were found to be around 3.03 eV, 3.05 eV, and 2.56 eV, respectively. Thus, the synthesized samples are expected to absorb visible light.

The obtained bandgap energy values of the CuO and CdWO_4_ samples were further used to determine the edge position of the valence band (VB) and the conduction band (CB) in the respective sample. To estimate the edge position, the following theoretical formulas reported in the literature were used [16]:

E_VB_ = X-Ee + 0.5Eg, E_CB_ = E_VB_ − Eg

Here, E_VB_ and E_CB_ are the edge positions for the VB and CB semiconductors; Ee and Eg are the energy of free electrons (~4.5 eV) and the bandgap energy of the semiconductors, respectively; and X is the electro-negativity of the semiconductor. The X values of the CuO and the CdWO_4_ were obtained from the previous literature as 5.81 and 6.28 eV, respectively [16,45]. The E_VB_ values were calculated to be 2.82 and 3.30 eV, and the E_CB_ values were estimated to be −0.20 and 0.255 eV, for the CuO and the CdWO_4_, respectively. These results strongly reveal that the heterojunction between CuO and CdWO_4_ can be effective in preventing the recombination of photogenerated electrons and holes.

### 3.6. Photoluminescence (PL) Analysis

**Figure 7:**
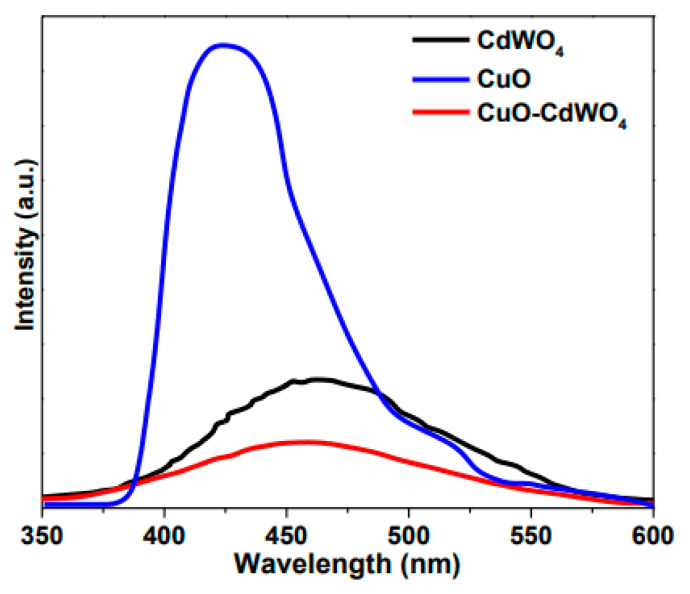
Photoluminescence (PL) spectra for CuO, CdWO_4_, and CuO–CdWO_4_ samples

Photoluminescence (PL) spectra were obtained to elucidate the migration and separation efficiency of photo-generated charge carriers at the interface of the CuO–CdWO_4_ semiconductor. The PL emission is the result of electron–hole (e^−^-h^+^) recombination in the semiconductors [16]. Accordingly, the e^−^-h^+^ recombination rate is considered to be directly proportional to the PL intensity. A lower PL intensity reveals a lower e^−^h^+^ recombination rate, thus suggesting the effective photocatalytic applications of the investigated photocatalyst [16]. The PL spectra for the CuO, CdWO_4_, and CuO–CdWO_4_ samples are shown in Figure 7. The samples exhibit PL emission in the visible region (400–500 nm). As can be seen in Figure 7, the CuO sample has higher PL intensity than that of the CdWO_4_ sample. This intensity decreases further in the CuO–CdWO_4_ composite. It can be deduced that the rate of e^−^-h^+^ recombination decreases significantly in the composite material, probably due to the synergetic effect between the CuO and CdWO_4_ semiconductors. This observation indicates that an improved photocatalytic behavior could be expected for the CuO–CdWO_4_ sample compared to the pristine semiconductors. Besides, the PL spectra provide strong evidence for the interaction between CuO and CdWO_4_ [16,46].

### 3.7. X-ray Photoelectron Spectroscopy (XPS) Analysis

**Figure 8:**
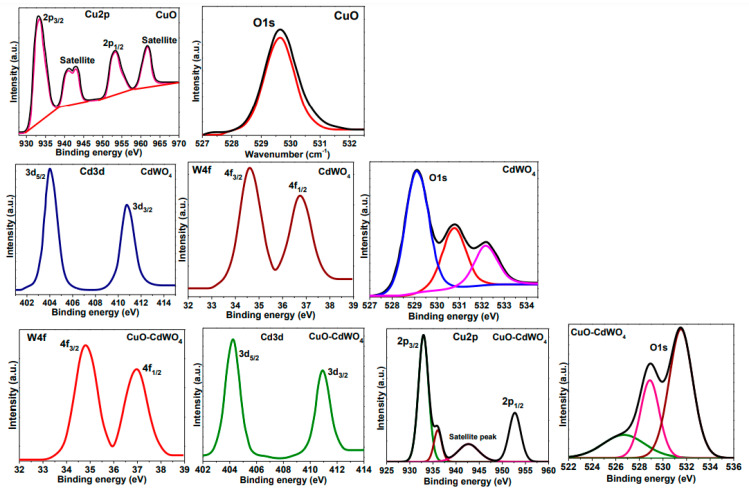
XPS analysis of CuO, CdWO_4_, and CuO–CdWO_4._

The synthesized samples were analyzed using XPS analysis to determine the surface properties of the samples. Comparative XPS analysis of the CuO, CdWO_4_, and CuO–CdWO_4_ samples was carried out and the changes in binding energy upon the interaction between CuO and CdWO_4_ were investigated (Figure 8). The XPS profiles confirm the coexistence of Cu, Cd, W, and O elements in the nanocomposite. The pristine CuO XPS spectra reveal the appearance of two characteristic peaks for Cu species with the spin energy separation of ~20 eV at 933.05 and 953.16 eV corresponding to the Cu2p_3/2_ and Cu 2p_1/2_, respectively [47]. Two satellite peaks also appear which could be assigned to the Cu^2+^ for the CuO sample. These peaks were identified following previous studies, where these peaks reveal the existence of Cu^2+^ oxidation in the CuO NPs [47,48]. The fitting of the O1s XPS spectra (for CuO) reveals two identical peaks at ~529.0 eV, suggesting the different environment of oxygen in the CuO sample [47]. The pristine CdWO_4_ XPS spectra for Cd ions appear with two characteristic peaks corresponding to the Cd3d_5/2_ and Cd3d_3/2_, with the spin energy separation of ~6.70 eV, at 405.58 eV and 412.32 eV, respectively, suggesting the existence of Cd^2+^ state in the CdWO_4_ sample [16]. The W 4f XPS spectrum indicates two characteristic peaks of the W element (+6 oxidation state in CdWO_4_) located at 35.37 eV and 37.62 eV, respectively [49]. The fitting of the O1s XPS spectra (for CdWO_4_) indicates the presence of three binding energy peaks at ~529.0, ~531.0, and ~532.5 eV, revealing the binding energy for adsorbed oxygen, hydroxyl oxygen, and lattice oxygen in the CdWO_4_ sample, respectively [16,49]. After the coupling of CuO to CdWO_4_, the shifting in the binding energy is clearly seen in the corresponding XPS spectra of the CuO–CdWO_4_ sample. The shift in binding energy is due to the electron transfer between CuO and CdWO_4_, which is very favorable for the improved photocatalyst application of CuO–CdWO_4_. The fitting of the O1s XPS spectrum for the CuO-CdWO_4_ sample results in the appearance of binding energies for O species from CuO, CdWO_4_, and adsorbed H_2_O [48,49]. Therefore, the XPS results confirm the formation of CuO–CdWO_4_ via a strong interaction between CuO and CdWO_4_, and these findings are consistent with other analytical results.

### 3.8. Photocatalytic H_2_ Production over Synthesized Photocatalysts

**Figure 9:**
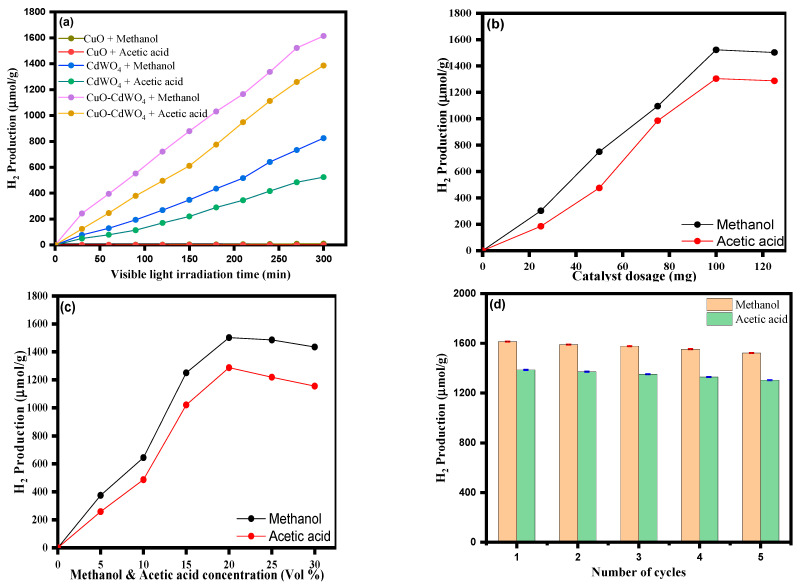
Production of H_2_ as a function of (**a**) visible light irradiation time (catalyst concentration = 100 mg, methanol = 20 vol.%, and acetic acid = 20 vol.%), (**b**) catalyst dosage (methanol = 20 vol.% and acetic acid = 20 vol.%), (**c**) methanol and acetic acid concentration (catalyst concentration = 100 mg) and (**d**) H_2_ production for five cyclic runs (catalyst mass = 100 mg, methanol = 20 vol.%, and acetic acid = 20 vol.%).

The synthesized nanomaterials were investigated as photocatalysts for H_2_ production via water splitting in the presence of visible light radiation. The photocatalytic investigations were achieved with the addition of 100 mg of photocatalyst in pure water under visible light irradiation for 5 h. The experiments were also performed using methanol or acetic acid (20 vol.% in water) as a sacrificial agent for H_2_ production [50]. The obtained results over the CuO–CdWO_4_ nanocomposite were compared with the H_2_ production ability of the pristine CuO and CdWO_4_ under the same reaction condition, as depicted in Figure 9a–d The results in Figure 9 clearly reveal a remarkable increase in H_2_ production efficiency in the case of the CuO–CdWO_4_ nanocomposite in the presence of methanol as a sacrificial agent in comparison to the pristine CuO and CdWO_4_ at any given reaction time. The observed results of photocatalytic H_2_ production under visible light ascribe that effective charge separations were attained when the CuO NPs were embedded on the surface of the CdWO_4_ [10,50]. The DRUV-vis and PL spectral analyses confirmed this claim. Clearly, the sacrificial agent played a significant role in the water-splitting process [50], as the highest rate of H_2_ production was achieved in the case of methanol rather than acetic acid.

Figure 9b shows the influence of catalyst dose on the photocatalytic H_2_ production over the CuO–CdWO_4_ catalyst using 20 vol.% sacrificing agents. The H_2_ production enhanced gradually with an increase of catalyst weight up to 100 mg, however, further increase could not alter the H_2_ production ability of the catalysts. The observed results indicated that a 100 mg catalyst dose was optimal to obtain the best photocatalytic performance; this was probably because the number of catalytically active sites exposed to reacting molecules did not change when the catalyst amount was higher than 100 mg. It was also possible that visible light could not reach the active sites as an increase in light scattering occurred with an increase in catalyst dose. The H_2_ production increased with the increase of vol% of methanol or acetic acid and reached its highest at 20 vol.% in the case of the CuO–CdWO_4_ nanocomposite [Figure 9c]. However, a further increase in vol% of methanol resulted in a slight decrease in H_2_ production. Similar behavior was observed in case of other photocatalysts [9]. An increase in the vol% of the sacrificial agent resulted in the adsorption of a greater number of methanol or acetic acid molecules on the semiconductor surface, blocking visible light from reaching the surface of the catalyst and resulting in a decrease in the H_2_ production rate.

The reusability of the CuO–CdWO_4_ nanocomposite was tested for five consecutive cycles. The experiments started with the CuO–CdWO_4_ mass of 100 mg and 20 vol% of methanol as a sacrificial agent at 25 °C, as mentioned in Figure 9d. After each cycle, the catalyst was recovered as a white powder using centrifugation, followed by washing with deionized water and drying using N_2_ purging at room temperature. The XRD and TEM analyses (Figures S1 and S2, ESI) of the spent catalyst confirmed the stability of the CuO–CdWO_4_ nanocomposite as no changes of morphology and phase structure, except the partial loss of crystallinity, were observed. It is clear from Figure 9d that, after consecutive five cycles, the H_2_ production is at a much higher rate in the CuO–CdWO_4_ in methanol compared to acetic acid, which retains 90% and 80% of its initial catalytic activity, respectively. It is worth mentioning that, without the sacrificial agents, the catalyst did not produce any considerable H_2_; while in the presence of methanol and acetic acid, a complete H_2_ production was achieved after each consecutive catalytic run. The slight decrease in efficiencies could probably be due to the loss of nanocomposite catalyst during the recovery and drying process and might be attributed to the passivation of the nano-catalytic active surface by increasing the concentration of intermediate charge carriers.

### 3.9. Proposed Reaction Mechanism of H_2_ Production over CuO–CdWO_4_

**Figure 10:**
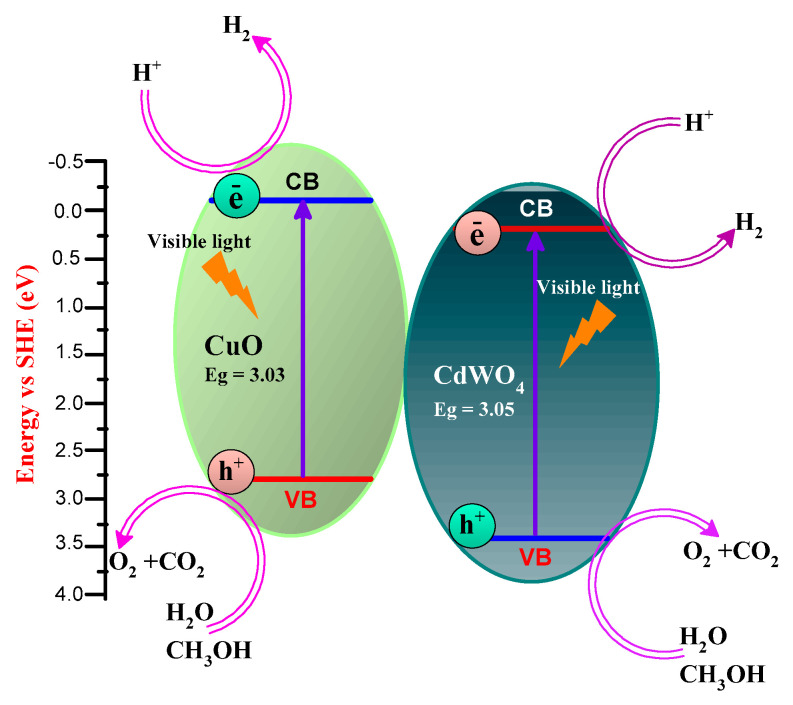
Proposed reaction mechanism of H_2_ production using sacrificing reagents in the presence of CuO–CdWO_4_ nanocomposite.

The possible mechanism for the interfacial charge transfer in the CuO–CdWO_4_ sample under visible light irradiation is proposed in Figure 10. When the CuO–CdWO_4_ is exposed to visible light, the photogenerated electrons are transported from the VB to the CB of the CuO–CdWO_4_ [10,50]. As shown in Figure 10, there is a possibility of the generation of e^—^ and h^+^ pairs in both CuO and CdWO_4_ semiconductors. According to the previous literature, when two different semiconductors (such as CuO and CdWO_4_) are conjugated, a realignment of the Fermi level generally occurs to meet the thermal equilibrium [10,50]. Since the CB of CuO is more negative than the CB of CdWO_4_, in the case of the CuO–CdWO_4_ composite, the electrons can rapidly transfer from the CB of CuO to the CB of CdWO_4,_ which leads to the realignment of the Fermi level until the thermal equilibrium is achieved [27]. Meanwhile, the photogenerated h+ from the highly positive VB of CdWO_4_ is immigrated to the less positive VB of CuO. This results in the redistribution of electric charges across the semiconductor interface and the formation of the Schottky barrier [27]. The electrons can be trapped in the barrier, and this can lead to the prevention of the recombination of charge carriers. The produced e- and h+ diffuses and reaches the surface of the CuO–CdWO_4_ composite and reacts with the water molecules (and sacrificial agents) adsorbed on the surface of the catalyst. It should be further noted that the edge positions of the CB and the VB define the e- and h+ reaction ability, respectively. In the case of water splitting, the photoreduction of water occurs by the photogenerated e-. In this case, the bottom of the CB must be more negative than the H+/H_2_ redox potential (0 eV vs. NHE, pH 0) [50]. The photooxidation reaction is carried out by h+, for which the top of the VB must be more positive than the oxidation potential of O_2_/H_2_O (1.23 eV vs. NHE, pH 0) [50]. As can be seen in Figure 10, the edge positions of the CB and the VB for the current material (CuO–CdWO_4_) meet the required criteria, thus the CuO–CdWO_4_ could be suitable for water splitting via a redox reaction. In addition, the methanol used in the photo-reforming process can enhance the efficiency of H_2_ production. Sacrificial agents, which have lower oxidation potential (for instance, 0.45 V vs. NHE for methanol), may easily and more efficiently scavenge the h+, thus largely preventing the e^−^-h+ recombination. The photooxidation of methanol can be carried out through scavenged h+; therefore, the sacrificial agents may also serve as proton sources. Therefore, the use of methanol can be considered as the coupling of the oxidation of an organic substance and proton reduction. Herein, the CuO–CdWO_4_ composite showed enhanced photocatalytic activity for water splitting when methanol was used as the sacrificial agent. This result is supported by previous studies [10,50].

## 4. Conclusions

In conclusion, the bare CuO, CdWO_4_, and CuO–CdWO_4_ nanocomposite samples were prepared using a simple facile green chemistry-based approach. Different analytical techniques, such as XRD, FT-IR, DRS UV-*vis*, SEM, TEM, N_2_ physisorption, PL, and XPS, were used to analyze the physicochemical properties of the prepared samples. In addition, the synthesized samples were utilized as catalysts for photocatalytic H_2_ production under visible light irradiation. The role of different reaction conditions, including visible light irradiation time, catalyst mass, concentration of sacrificial reagents, and reusability for five cycles, was studied. The maximum H_2_ was observed while using methanol as a sacrificial agent, which was highest when used over the CuO–CdWO_4_ nanocomposite. This enhancement was due to the faster charge separation and higher visible light absorption as a result of the synergistic effect between the CuO–CdWO_4_nanocomposite and methanol. A new method for the synthesis of CuO-CdWO_4_ nanocomposite was revealed. The photocatalytic study showed an increase in the activity of CuO–CdWO_4_ towards methanol for H_2_ generation. The reusability study clearly showed the stability of the developed photocatalyst. The present synthesis approach could be very useful to develop a highly stable photocatalyst under visible light irradiation.

## Figures and Tables

**Table 1:** FT-IR analysis of CuO, CdWO_4_, and CuO–CdWO_4_ samples.

CuO		CdWO_4_		CuO–CdWO_4_	
Wavenumber Region (cm^−1^)	Assigned Group	Wavenumber (cm^−1^)	Assigned Group	Wavenumber Region (cm^−1^)	Assigned Group
3398	-O-H stretching	3419	-O-H stretching	3481–3400	-O-H stretching
2060	C=N stretching	2923 and 2853	Stretching vibration of C-H of CH_3_, and CH_2_, respectively	-	-
1590	C=O and/or -NH stretching	1744–1639	C=O, -NH, and aromatic C=C double-bond functional groups	1670–1590	C=O, -NH, and aromatic C=C double-bond functional groups
1400–1023	Aromatic C–C groups and C–O functional groups	1460–1020	Aromatic C–C groups and C–O functional groups	1460–1020	Aromatic C–C groups and C–O functional groups
935–700	Deformation vibration of C–H bonds	-	-	-	-
600–400	Cu–O bond	900–500	Intrinsic vibrations from CdWO_4_	1000–400	Vibrations from CuO and CdWO_4_

**Table 2:** EDX analysis of CuO, CdWO_4_, and CuO–CdWO_4_ samples.

Order	Elements	EDX Analysis (Atom %)		
		CuO	CdWO_4_	CuO–CdWO_4_
**1.**	Cu	28.33	-	38.24
**2.**	Cd	-	20.84	14.05
**3.**	W	-	45.32	24.85
**4.**	O	53.16	33.86	22.85

## Data Availability

Not applicable.

## Supplementary Materials

The following supporting information can be downloaded at: https://www.mdpi.com/article/10.3390/nano12244472/s1, Synthesis methodology for CuO and CdWO_4_ nanoparticles using extract of the *Brassica rapa* plant; Figure S1: XRD patterns for CuO-CdWO_4_ composite (a) fresh and (b) spent for 5 cycles; Figure S2: TEM images for CuO-CdWO_4_ composite (a) fresh and (b) spent for 5 cycles

## Appendix A

This study is an extension of our previous studies [22,27], therefore, we obtained the copyright permission from the publishers.
