# Supplementary material for: One-Pot Facile Synthesis of CuO–CdWO4 Nanocomposite for Photocatalytic Hydrogen Production
Source: Nanomaterials (Basel). 2022 Dec 16;12(24):4472. doi: 10.3390/nano12244472 (PMC9782073; doi:10.3390/nano12244472)
Supplement: Supplementary file 1 [file nanomaterials-12-04472-s001.zip › nanomaterials-2025136-supplementary.pdf]

nanomaterials

MDPI

Supplementary Materials

One-Pot Facile Synthesis of CuO–$CdWO_4$ Nanocomposite for Photocatalytic Hydrogen Production

Shaeel Ahmed Althabaiti [1], Maqsood Ahmad Malik [1], Manoj Kumar Khanna [2], Salem Mohamed Bawaked [1], Katabathini Narasimharao [1,*], Soad Zahir Al-Sheheri [1], Bushra Fatima [3,] and Sharf Ilahi Siddiqui [3,4,*]

[1] Chemistry Department, Faculty of Sciences, King Abdulaziz University, P.O. Box 80203, Jeddah 21589, Saudi Arabia
[2] Department of Physics, Ramjas College, University of Delhi, Delhi 110007, India
[3] Department of Chemistry, Jamia Millia Islamia, Delhi 110025, India
[4] Department of Chemistry, Ramjas College, University of Delhi, Delhi 110007, India
* Correspondence: nkatabathini@kau.edu.sa (K.N.); sharf_9793@rediff.com (S.I.S.)

Synthesis of CuO and $CdWO_4$ nanoparticles using extract of the *Brassica rapa* plant

Plant extract: Initially, *Brassica rapa plant* extract was prepared. The collected green *Brassica rapa* leaves were washed with deionized water several times to remove any dust, leachable impurities, then the leaves were grounded to a thick paste using a mortar pestle. Then, 10 g of the obtained paste was poured into 100 mL of distilled water, and it was heated on water bath at 50°C for 2 h, and then the resulting extract was filtered using Whatman paper.

CuO: In brief, 25 mL of aqueous *Brassica rapa* plant extract was mixed with 20 mL of 0.2 mol L^{-1} copper nitrate solution, and to this 0.1 mol L^{-1} urea solution was added, followed by addition of 10 mL of 1.0 mol L^{-1} NaOH solution. The mixture was stirred at 50°C for 45 min and after that a blue color precipitate was obtained, which indicate the formation of CuO NPs. The colored precipitate of CuO NPs was centrifuged at 1500 rpm for 10 min, washed with distilled water several times and the obtained product was dried at 80°C for 24 h.

$CdWO_4$: To prepare $CdWO_4$, 60 mL of the extract was added slowly under continues stirring to a solution mixture containing 300 mL of 0.2 M sodium tungstate solution and 100 mL of 0.2 M cadmium iodide solution. The reaction occurred between sodium tungstate and cadmium iodide leading to the formation of $CdWO_4$ and NaI. The obtained precipitate was then washed with distilled water several time, centrifuged at 8000 rpm for 5 min, dried at 80°C for 8 h and then calcined at 200°C for 2h in a muffle furnace under static conditions.

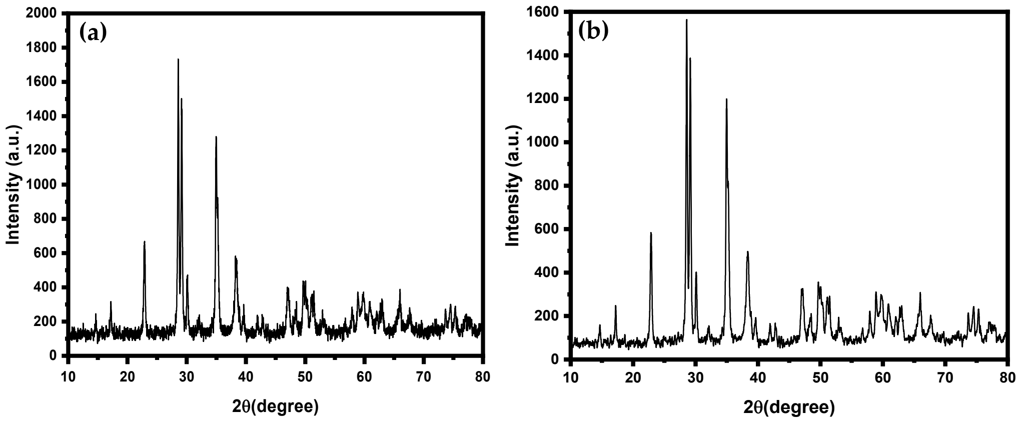

Figure S1. XRD patterns for CuO-CdWO4 composite (a) fresh and (b) spent for 5 cycles.

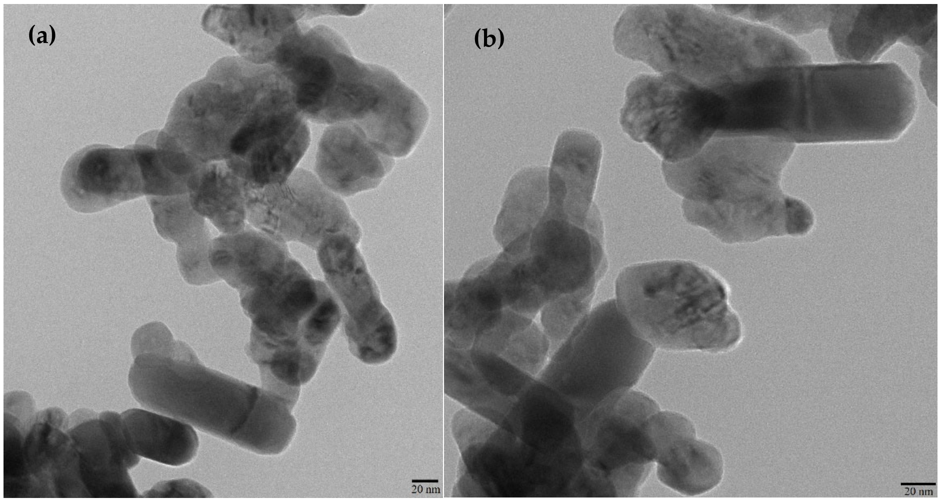

Figure S2. TEM images for CuO-CdWO4 composite (a) fresh and (b) spent for 5 cycles.
